# Spatial Ecology and Diel Activity of European Wildcat (*Felis silvestris*) in a Protected Lowland Area in Northern Greece

**DOI:** 10.3390/ani11113030

**Published:** 2021-10-21

**Authors:** Despina Migli, Christos Astaras, George Boutsis, Anastasia Diakou, Nikolaos-Evangelos Karantanis, Dionisios Youlatos

**Affiliations:** 1 School of Biology, Aristotle University of Thessaloniki, 54124 Thessaloniki, Greece; despmigk@bio.auth.gr (D.M.); gboutsis@bio.auth.gr (G.B.); 2 Forest Research Institute, Hellenic Agricultural Organization “DEMETER”, 57006 Vasilika, Greece; christos.astaras@fri.gr; 3 School of Veterinary Medicine, Aristotle University of Thessaloniki, 54124 Thessaloniki, Greece; diakou@vet.auth.gr; 4 The Princess Royal College of Animal Management and Saddlery, Capel Manor College, Enfield EN1 4RQ, UK; nekarantanis@gmail.com

**Keywords:** European wildcat, telemetry, accelerometer, home range, activity pattern, ODBA, Greece

## Abstract

**Simple Summary:**

The European wildcat is a species of conservation concern protected across its range in Europe, where it occurs in five discontinuous populations. The Balkan population has received little attention, making it difficult to assess whether the ecological traits reported for other populations apply also to this population. This hampers the development of targeted conservation measures. The present study reports the first findings on the spatial ecology and daily activity pattern of wildcats in a human modified landscape in Greece, using cutting edge data loggers attached to wildcat collars. In Greece, wildcat home range sizes are within the range of those reported for other populations. Male wildcats are active primarily at night and near dawn and dusk, as is typical for the species. However, the activity of some females varied from this pattern in late spring, in ways expected for wildcats, caring for offspring at a den. Overall, our findings help fill the ecological knowledge gap of the species in Greece and suggest that lowland agricultural areas with patches of natural habitats may have a significant role in the future conservation of the species.

**Abstract:**

The Balkan populations of the European wildcat are among the least studied. This study reports the first findings on the spatial ecology and activity pattern of the wildcat in Greece and compares them to those of better studied northern populations. We fitted five wildcats (two males, three females) with collars containing GPS and accelerometer loggers (E-obs 1A) and collected data from fall to early summer. All animals moved within a mosaic of lowland agricultural fields, woodland patches, riparian forests and wetlands near the banks of a lake. The trapping rate was the highest reported for the species. The home range sizes, estimated using Brownian bridge movement models, ranged from 0.94 to 3.08 km^2^ for females and from 1.22 to 4.43 km^2^ for males. Based on overall dynamic body acceleration (ODBA) values estimated from the accelerometer data, the diel activity of male wildcats followed the species’ typical nocturnal pattern with crepuscular peaks. Female activity varied seasonally, at times being cathemeral. We found only weak effects of environmental variables on wildcat activity, and no significant difference in the activity in open versus forested areas. Our findings suggest that human modified landscapes can play a significant role in the conservation of this typically forest-associated species.

## 1. Introduction

**Figure 1:**
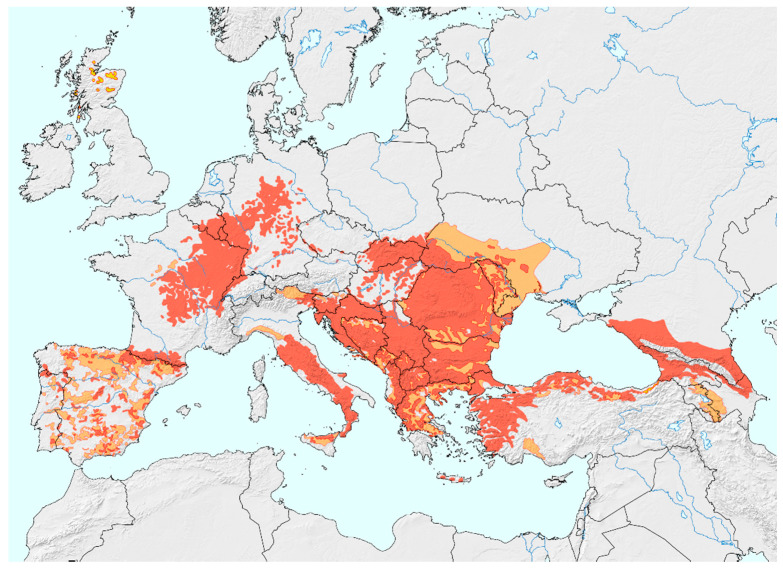
Current distribution of the European wildcat (*Felis silvestris*). Coloured areas: red = present, orange = possibly present, yellow = possibly extinct. Used after permission by Peter Gerngross [1].

The European wildcat (*Felis silvestris*), hence “wildcat”, is a small carnivore with a wide but discontinuous distribution (Figure 1) [1,2]. While IUCN classifies the species as one of “Least Concern” [3], several populations are declining due to threats such as habitat fragmentation, [4,5,6] human-induced mortality [7,8,9] and hybridisation with the domestic cat (*Felis catus*) [10,11]. In Europe, five main biogeographic wildcat groups have been identified, supported by recent genetic studies: Iberian Peninsula, central Germany, (western) central Europe, Eastern Alpine (Italian Peninsula) and southeast Europe (Dinaric Alps and the Balkans) [12,13]. Among these, the Balkan populations have received the least scientific attention to date. Although Romanian populations appear stable [14], the National Red Data Books list the wildcat as endangered in Bulgaria ([15]), near threatened in Kosovo [16] and data deficient in Greece [17].

The sole publication on wildcat distribution and ecology in Greece was three decades ago [18]. The study reported the species’ presence at 40 sites (0 m to 1500 m altitude), primarily in mainland Greece, based on direct observations, tracks and dead animals. Habitats used included pine, oak and birch, and riparian forests, maquis, wetlands, semi-intensive agricultural areas and orchards. Since then, information on the species distribution and conservation status has been opportunistic, e.g., as by-catch detections in camera trap surveys aimed at other species, and available only in hard to access grey literature reports. 

Understanding a species’ spatiotemporal activity patterns is key baseline knowledge for assessing its susceptibility to current or future threats, and for designing efficient population monitoring protocols. For instance, home range size estimates could inform both conservation efforts to assess habitat availability and fragmentation [19,20], and the design of abundance surveys employing spatially explicit capture-recapture methods (e.g., by ensuring that >1 camera is within an average home range; [21,22]. Likewise, knowledge of wildcat diel and seasonal activity patterns can help assess temporal overlap with feral/domestic cats and human activities (e.g., hunting, road traffic), and therefore provide insight on possible underlying ecological processes increasing the risk of hybridization and anthropogenic mortality (e.g., poaching, vehicle collisions) [9]. While some ecological traits of the wildcat are universal across its range, such as its solitary and territorial nature (e.g., [8,19] and crepuscular/nocturnal activity [23,24] space use has been shown to vary widely. For example, home range size reports of both sexes vary by as much as a degree of magnitude across studies (males: 1.9–50.2 km^2^; females: 0.7–13.9 km^2^; [5,20]). These pronounced variations limit the confidence with which ecological knowledge from better studied populations can be transferred, untested, to less studied ones, like the wildcat populations in the Balkans. 

In this study, we report the first ever findings on the spatial ecology and activity pattern of the wildcat in Greece (and the Balkans, as far as we are aware) using GPS telemetry. In doing so, the study contributes towards bridging the current knowledge gap in the ecology of the species in southeast Europe. It is also one of the few studies to use very high temporal resolution accelerometer logged data to examine wildcat activity. Specifically, the aims of the study were to:

(a) estimate the home range of two male and three female wildcats in Koronia and Volvi Lakes National Park (NPKV) in northern Greece,
(b) describe the diel activity of both sexes,
(c) identify potential environmental variables (e.g., weather, day length, moon phase) significantly affecting wildcat activity,
(d) evaluate whether non-resting wildcat activity differs in open vs. forested areas, and
(e) ascertain resting wildcat site fidelity across seasons.

Finally, by nature of our study area being in a wetland-agricultural mosaic, a landscape where the species appears to have one of the highest densities in Greece, our findings add to increasing evidence challenging the once accepted notion that the wildcat is primarily a forest resident of broad-leaved or mixed forests ([3]). Our study further investigates the importance of open-closed mosaic environments, where both hiding and hunting sites are available, for the species’ survival [8,25]. Their importance has been previously demonstrated in other Mediterranean areas [20,26,27] and recently in central Europe [28] for shrubland-pasture landscape mosaics.

## 2. Materials and Methods

### 2.1. Study Area

**Figure 2:**
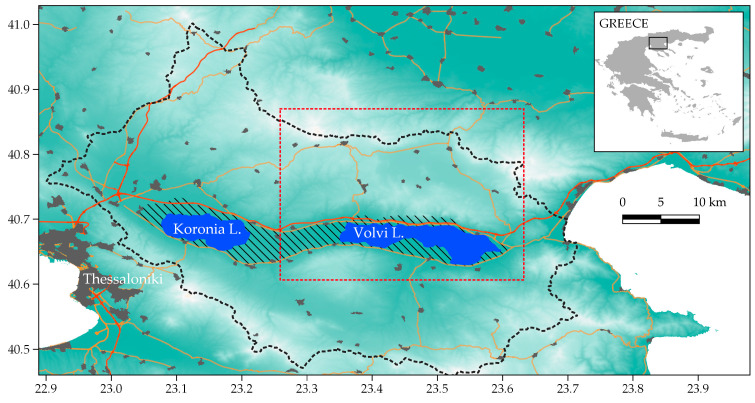
Map depicting the boundary of the Koronia-Volvi lakes National Park (dotted black line) and core zone (dashed area; Natura 2000 area GR1220001). Wildcat trapping was conducted near the southern banks of Lake Volvi, within the core area. [Grey areas mark urban areas incl. the metropolitan area of Thessaloniki, red lines motorways, orange lines primary and secondary two-way roads, and red dotted polygon the area within which the collared wildcats moved during the study period].

This study was conducted within the core zone of Koronia and Volvi Lakes National Park (NPKV) (41° N; 23.12° E to 40.46° N; 23.52° E; 21.200 km^2^, Figure 2), an important Ramsar wetland in northern Greece. The core zone is since 2011 included in the European Union’s Natura 2000 network of protected areas as a Special Area of Conservation (GR1220001; 288.3 km^2^, 560 m a.s.l). About 57.1% of the area is agricultural land, consisted mainly of winter cereals, alfalfa and maize fields. Natural habitats consist primarily of reed beds and humid grasslands around the lakes, riparian gallery forests and thickets along the many rivulets and intermittently flowing rivers draining into the lakes, and a mosaic of small forest patches (*Quercus ilex* and *Q. rotundifolia*), hedges and tree lines. The climate is typical Mediterranean. Annual rainfall ranges from 400 to 450 mm, distributed almost entirely during the winter. Winter flooding and summer droughts are common [29]. Daily temperature ranges from −10 °C to 17 °C in the winter and 12 °C to 42 °C in the summer. To the north and south of the lakes the terrain is mountainous (up to 1.165 m a.s.l.) and rugged. A motorway (fenced) runs east-west north of the lakes, as does a high speed and traffic (dual-way) road to the south (Figure 2). There are several small villages within and in the periphery of the study area, with human activities being mostly agricultural. Earlier camera trapping by the authors (D.M., G.B.) has confirmed the presence of wild boar, stone marten, badger, otter, wolves and dogs.

### 2.2. Trapping and Handling

**Figure 3:**
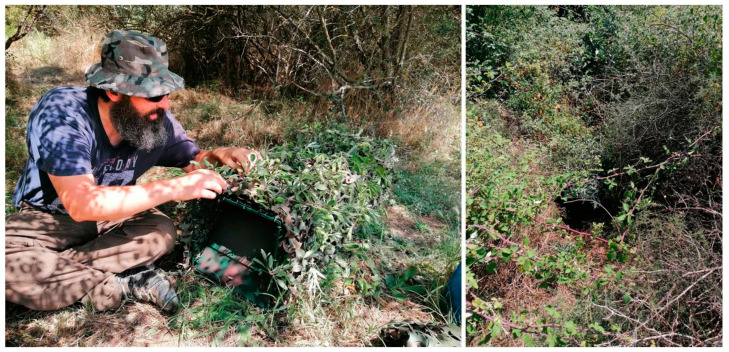
Preparation and placement of the cage traps used.

Trapping took place from September to November 2020. During the preceding month, we identified passages through dense vegetation (e.g., bramble) that were regularly used by wildcats, using trail cameras and frequent surveys for felid feces and tracks. Eight two-door live animal cage traps (102 × 32 × 35 cm) were set along such passages so that an animal could pass, without a detour, only by walking through the trap. To avoid attracting other mesocarnivores (e.g., stone marten *Martes foina*), animal baiting was avoided. Moreover, an earlier six-month study in the same National Park by the authors showed that wildcats did not appear attracted to hair trap sticks treated with valerian root, based on examination of footage from camera traps aimed at the hair trap stations (unpublished data). Therefore, we decided to proceed without any bait, especially since unbaited wildcat trapping had been previously proven possible [30]. All cage traps were carefully camouflaged with military, cameo-colored, net and natural vegetation to match the surrounding environment. If required, branches were placed at the flanks or on top of the traps to block animals passing around the trap. (Figure 3). Given the quick success of this trap setting, we did not further consider baiting the traps.

**Figure 4:**
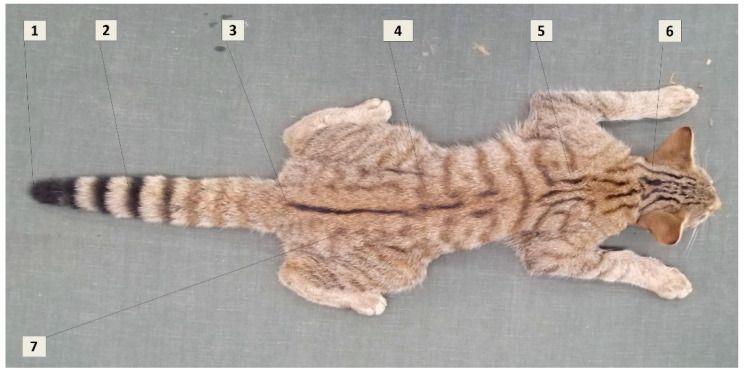
The seven key pelage characteristics of the European wildcat (1) Shape of tail tip: blunt (2) Distinctness of tail bands: Distinct—not fused or joined with dorsal line (3) Extent of dorsal line: stops at base of tail (4) Broken stripes on flanks and hindquarters: <25% broken (5) Stripes on shoulder: Two thick stripes (6) Stripes on nape: four thick stripes (7) Spots on flanks and hindquarters: none [33,34].

Traps were checked daily. Ketamine (5 mg/kg) with medetomidine (40 μg /kg), IM was administered to the wildcats to produce the required sedation. Recovery was expedited by the administration of atipamezole (100 μg/kg), IM. All animals were released only after having recovered and being fully alert. We recorded the following information for each animal: gender, weight, morphological measurements and approximate age (juvenile = 0–12 months; subadult = 12–24 months; adult >24 months) as estimated based on body size, tooth wear and—for females—the presence or not of pre- or post-reproductive teats [31,32]. We also collected blood samples for a concurrent parasitological study and to confirm genetically the phenotype-based identification of each animal as a European wildcat. Morphological identification and assignment to the European wildcat were performed based on coat pattern markings as per Kitchener [33] (Figure 4).

The research met all the welfare and ethical criteria concerning research involving wildlife set by the General Directorate of Forests and Forest Management (Hellenic Ministry of Environment and Energy; Research Permit Nr. 8817/242/29 May 2020).

### 2.3. GPS Collaring

Captured wildcats were fitted with 69 gr collar-mounted global positioning system (GPS) and tri-axial accelerometer (ACC) loggers and a UHF radio transmitter (e-obs 1A, e-obs GmbH, Grünwald, Germany). The collars accounted for less than 3% of the animal’s body mass (range: 2.6–3.7 kg) as per the recommendations of Kenward [35] The loggers recorded position at 30 min intervals (48 waypoints/day) and acceleration on three axes in 4 sec bursts every 2 min (10 Hz). When accelerometer readings exceeded 10,000, the position interval was halved to capture in higher spatial resolution the animals’ travel route. The data were stored on board the collar until they were transferred to a base station (e-obs GmbH, Grünwald, Germany) every 1–2 weeks via a VHF connection from up to 2 km. We searched for the UHF signal of each collar using an AR8200 (AOR Ltd., Tokyo, Japan) receiver with a 10-element Yagi antenna, in order to approach the animals enough to establish a data transfer connection. In order to reduce the daily energy consumption of the loggers, data transfer was enabled only for a 2 h window each day.

### 2.4. Home Range Estimation

Wildcat space use was assessed using the Brownian bridge movement model (BBMM), a home range (HR) estimator that uses animal movement paths rather than discrete locations to quantify the relative intensity of use of a landscape [36] Movement paths are estimated empirically using the distance and relatively short time interval between successive pairs of locations. A BBMM assumes that locations are not independent, and therefore is ideal for the temporally auto-correlated data obtained from GPS telemetry studies like ours. 

We did not consider locations taken during the first 24 h after releasing the GPS collared animals, to reduce the chance of including atypical movement patterns related to the capture and handling process. For all animals, we used the following model parameters in order to account for the spatial autocorrelation and locational error of the telemetry data: 15 min time step, 120 min maximum time lag between successive locations and 20 m standard deviation of location error (assuming normal distribution; estimated rounding up the 15.4 m horizontal inaccuracy of the raw collar data). Wildcat utilization distributions were constructed at 50 m resolution. We considered as HR the area encompassed by the 95% contour. We calculated both seasonal (October–December, January–March, April–June) and total HRs, depending on the data available for each animal.

To facilitate comparison of our results with earlier studies, we also calculated total and seasonal HRs using the 95% minimum convex polygon (MCP) estimator. Home range models were developed with R [37] packages *BBMM* [38] and *adehabitatHR* [39].

### 2.5. Activity Pattern Analysis

We calculated the overall dynamic body acceleration (ODBA) for each 2 min interval of the tri-axial accelerometer data using the Movebank Acceleration Viewer (v.34; [40]). ODBA has been used as a proxy of an animal’s energy expenditure [41] providing very high temporal resolution on animal activity that GPS data alone cannot provide with 15–30 min intervals between locations. This is especially true for a small animal like the European wildcat, where displacements can at times be small, making differentiation between resting and foraging location clusters challenging. 

To examine the diel activity of the wildcats, we calculated hourly ODBA values by averaging all 2 min interval values of any given hour. To examine activity patterns across the study period, we averaged the hourly ODBA values at the 24 h and nighttime (6:30 pm–6:30 am) level. We used a series of generalized mixed effect models (identity link function, constant error distribution) in R package *lme4::lmer* [42] to test how daily temperature (°C; minimum, maximum or mean), precipitation (mm), wind speed (m/s), day length (proportion of 24 h with daylight) and moon phase (0.00–1.00) affect wildcat 24 h or nighttime activity. We examined that the residuals of the models were normally distributed, and that there was a constant error distribution (homoscedasticity) using informal diagnostics in R. Weather measurements were obtained from a weather station located within the NPKV near Lake Koronia, approximately 30 km away. Predictive variables were standardized when not in 0–1 scale. (z-score, subtracting from each value the mean and dividing by SD). Each model accounted for individual wildcat differences by including animals as a nested random effect. We used Akaike Information Criteria (AIC) [43] for model selection. Variables were included in multivariate models only when they were informative (i.e., their univariate model had <AIC than the intercept only model). Among correlated variables, we kept for further consideration the one with the lowest univariate model AIC. Once a final set of fixed variables was selected, we run all possible multivariate combinations using *MuMIn:dredge* [44], using again AIC for model selection. The p-values for fixed effects were calculated using the Satterthwaite approximation in *lmerTest* [45]. Due to the large sample size and random effects, we used *p* = 0.001 as our significance threshold. The goodness-of-fit of the best model was assessed by calculating the coefficient of determination (R^2^) using R code provided by Byrnes [46], which calculates the correlation between fitted and observed values.

To examine whether non-resting wildcat activity differed between open and forested areas, we first assigned 2 min ODBA values to the temporally closer GPS location. Then, we calculated the mean ODBA of each location using only values up to 15 min before or after the time the GPS location was recorded (i.e., for a 10:00 am GPS point, we used ODBA values from 9:45 am to 10:15 am). Locations with less than 13 ODBA values (max. 15) were excluded from the analysis. We then removed resting locations, which we considered those where (a) mean ODBA + 2^x^SD ODBA was <2000, and (b) maximum 2 min ODBA value recorded in the 30 min period was <2000. Based on the observations we made of collar-fitted, resting wildcats prior to their release, as well as the hourly ODBA values from the diel activity analysis, we are confident that the criteria used to identify GPS locations where the animal had been resting for at least 30 min are robust.

We constructed generalized mixed effect models (identity link function, constant error distribution) in *lme4::lmer* [42] with mean ODBA of wildcats at non-resting GPS locations as response variable, % forest cover and darkness (0–1; 30 min after sunset up to 30 min before sunrise) as predictive variables, and individual wildcat as a nested random effect. We used the same model diagnostics, model selection criterion (AIC), p-value calculation and significance threshold, and coefficient of determination (R^2^) calculation as for the previously described 24 h and nighttime activity pattern analysis. Using Quantum GIS v.3.10.13 [47] and the Small Woody Features (SWF; 2015; 5 m resolution) and Tree Cover Density (2018; 10 m resolution) raster layers available at the European Union’s Earth observation program “Copernicus” [48], we estimated at 10 m resolution the percent forest cover within a 100 m radius from each GPS location, defining as forested areas with >20% TCD and/or SWF classification as hedgerow or small woodland patch. 

Finally, we identified the frequently used resting sites (>20 resting GPS locations within a 30 × 30 m area) of each animal per month. We examined whether these resting sites were the same across the study period.

## 3. Results

Overall, during 168 trap-nights, we trapped seven wildcats: three males and four females (trapping rate = 1 wildcat/24 trap-nights). Two were too small (weight and neck size) to be fitted with our GPS collars. The two males and three females we did collar were tracked for an average of 218 ± 66 days (range 99–261), with 10,185 ± 3114 fixes over a period of 274 days (Table 1). Due to our omission, the accelerometer logger was not activated in one female wildcat, and she was therefore excluded from the activity pattern analyses. In total, 580111 2 min accelerometer logs were recorded, with an average of 693 ± 24 per wildcat per day (90.9–99.8% of the 720 possible daily logs) (Table 1).

### 3.1. Home Range

**Figure 5:**
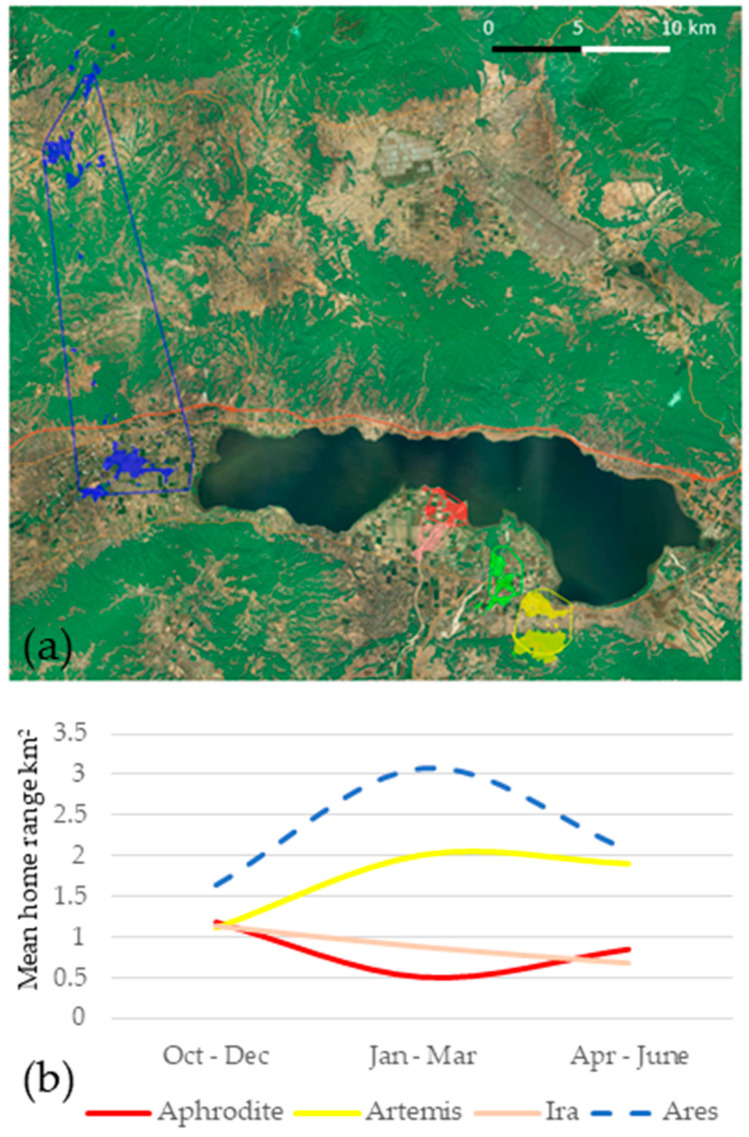
(**a**) Μinimum convex polygon (solid line) and brownian bridge (filled area) home ranges (95%) of the five GPS-collar fitted wildcats (male: Ares, Apollo; female: Aphrodite, Ira, Artemis) for the duration of the study period. [Background: Bing Satellite Image overlayed by green layer depicting forests and hedges] (**b**) Temporal variation of brownian bridge home range size of the four wildcats (Apollo is excluded as available data covered only October–December).

The overall home range size of female wildcats ranged from 0.94 to 3.08 km^2^ and of males from 1.22 to 4.43 km^2^, as estimated using the 95% BB home range contour (Table 2). MCP home range size estimates ranged from 1.69 to 4.79 km^2^ and 2.35 to 58.37 km^2^ for females and males respectively; an increase of 56–1200% over BB estimates. These differences are expected as MCP home ranges describe more the extent of distribution of an animal’s locations rather than its home range, and typically includes large areas never used by the animals [49]. They provide a value for comparison with older study findings, but MCP estimates—when viewed in conjunction with BB estimates—also capture the home range shift of animals within a given study period. In our case, the large MCP home range estimate of Ares captures this male’s travel of more than 25 km during the middle of the study period, and its subsequent return a couple of months later (Figure 5a). It is probably a similar move—also in the winter—by the other male, Apollo, that resulted in us losing track of the animal. We report only BB home range estimates in the rest of the results.

The variation in home range sizes was pronounced both across individuals and seasonally within individuals. Seasonal variation was significant in all wildcats, regardless of sex, but there was no evidence of universal temporal synchrony in these changes (Figure 5b). The two adult females, Aphrodite and Ira, reduced their home size in January–March and April–June periods compared to its peak in October–December, which could be related to denning behavior. On the contrary, the home range size variation of the subadult female, Artemis, was similar to that of the adult male, Ares. Like Ares, she also shifted her home range from lowland agricultural area to the slopes of wooded (maquis) hills during January–March. Moreover, by late spring Artemis made a 5 km linear southward excursion and subsequent return within 24 h, and an eventual return to her fall home range (lowland fields).

We did not observe home range overlap between the two female neighbouring animals, Ira and Aphrodite (Figure 5a), supporting the widely accepted territorial nature of the species (e.g., [8]). 

### 3.2. Activity Pattern

**Figure 6:**
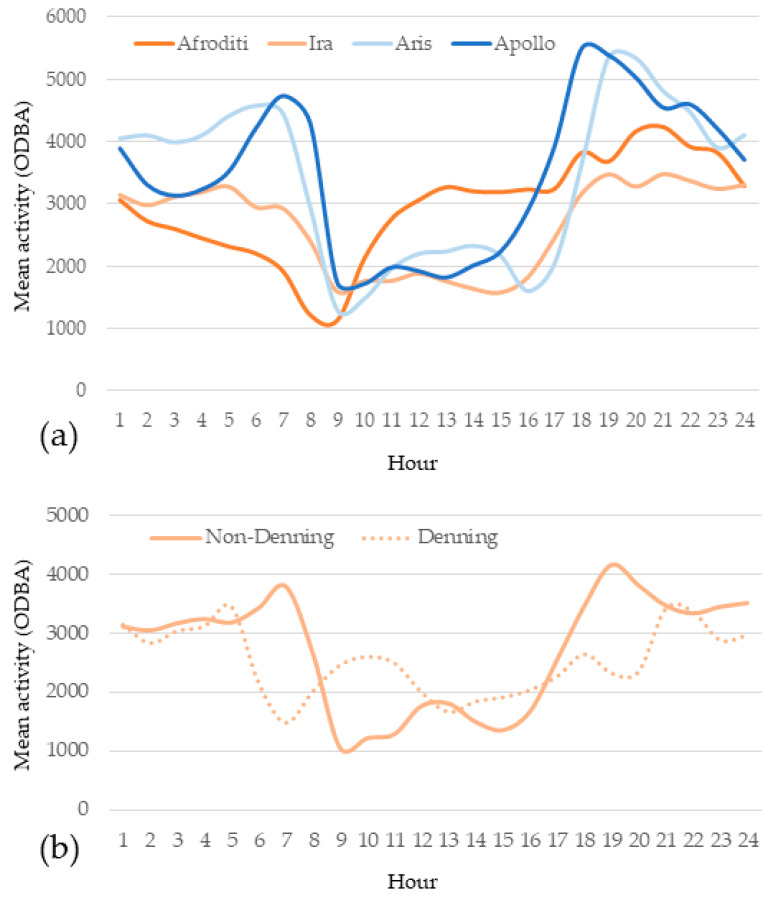
(**a**) Diel activity pattern of two male and two female wildcats based on ODBA values on a 24 h basis (**b**) Diel activity pattern of a female wildcat (Ira) during non-denning (October–March) and denning (April–June) periods, based on ODBA values on a 24 h basis.

The male wildcats were on average and across the study period more active than females, both when measured at the level of mean hourly ODBA (Ares 3393 ± 1249, Apollo 3481 ± 1185, Aphrodite 2943 ± 814, Ira 2645 ± 693; Figure 6a) and mean 24 h and nighttime ODBA level. Males also displayed a more typical nocturnal/crepuscular diel activity with pronounced activity peaks at dawn and dusk (Figure 6a). One female, Ira, displayed similar diel activity pattern as the males, but with a less pronounced crepuscular activity peak. This was however due to a more cathemeral activity during the April–June period (Figure 6b) when denning activity is known to occur. The diel activity during the non-denning period matches closely the one of the two males. On the contrary, the other female, Aphrodite, did not show such a seasonal variation in her diel activity pattern, which is best described as cathemeral, with a single resting period in early morning. This cathemerality persisted throughout the study period.

Two models with moon phase, day length and minimum temperature as predictors of mean ODBA activity at the 24 h level were best supported by our data (ΔAIC < 2) (Table S1). We therefore calculated the model average coefficients of these models (Table 3). The results indicate a significant but small negative relation of day length (i.e., proportion of 24 h with day light) and minimum temperature with wildcat activity. In the case of females, the effect of day length is reversed (Figures S1 and S2). Moon phase, though in the final set of variables, did not have a significant effect on wildcat activity of neither males nor females (Figure S3). The coefficient of determination of the global model (R^2^ = 0.271) suggests that these environmental variables explain only moderately the observed variation in 24 h level ODBA values. 

Our data supported one model for mean ODBA activity at nighttime level (ΔAIC < 2) (Table S2). Day length and minimum temperature were once again the sole significant informative variables (Table 4). The positive effect of minimum temperature observed at the 24 h activity models was four times stronger at the nighttime level, and observed for both sexes (Figure S1). Day length was negatively related to nightly ODBA, but this trend is only true for female wildcats (Figure S2). On the contrary, male wildcats increase their nightly activity during spring and summer. The coefficient of determination of the global model (R^2^ = 0.219) suggests again that these environmental variables explain only modestly the observed variation in nighttime level ODBA values. 

### 3.3. Resting Sites

We identified a total of 74 resting sites frequently used by the four wildcats for which we had accelerometer data (9–23 per animal; Table 5). Only a fraction of these sites were used in any given month (range 5–50%). In general, the wildcats rested throughout their range and invariably in forest patches and hedges. 

### 3.4. Activity Differences in Open and Forested Areas

The model including % forest cover (+ second order polynomial), darkness and interaction of % forest cover*darkness as predictors of wildcat activity at non-resting GPS locations (i.e., mean ODBA value for a period of 15 min before and after the GPS record) was the one best supported by our data (ΔAIC < 2) (Tables S3 and S4). However, the predictive value of this model is very low (R^2^ = 0.074), which means that most variation in the ODBA values remains unexplained. 

## 4. Discussion

Our study is the first systematic effort to describe the spatial ecology and diel activity of the European wildcat in Greece, and the first one using GPS telemetry within the Balkan/Southeast Europe range of the species. Although the study area was within the core of a protected area (NPKV), it is a human transformed landscape, where natural habitats are interspersed within intensively cultivated monocrop fields, villages and a busy high-speed motorway. Increasing our understanding of the role of such human-dominated landscapes for the persistence of the wildcat is important, especially in conjunction with recent range-wide efforts to examine the drivers of wildcat hybridisation [13] and human-caused mortality [9]; two major threats to the species [3]. 

The trapping rate at our site (4.17 wildcats per 100 trap-nights) is the highest reported both in Mediterranean habitats (Portugal: 1.59 [20], Italy: 0.26–0.48 [31,50]) and central Europe (Germany: 0.54 [32], Switzerland: 1.89 [51], Slovenia: 1.72 [30]). If the untested hypothesis of Anile et al. [31] that differences in trapping rates reflect underlying differences in wildcat density is true, then NPKV is home to one of the densest wildcat populations in Europe. Such an interpretation assumes similar trapping procedures were used across sites. A review of the studies cited above showed that, as far as we could deduce, all used same type of traps and the standard trapping procedures [30,50]. One difference is that we did not bait our traps, unlike some other studies that used either scent (e.g., valerian, [32]) or food (e.g., live birds, [20]) as lures. Baiting might increase trapping rate, so this difference is unlikely to explain the high trapping rate in NPKV. Another difference is that we chose our trap sites after a brief but intensive survey in the surrounding areas for wildcat signs (feces, tracks, camera trap detections). All other studies trapped in areas with confirmed wildcat presence, but it is unclear at what scale this information was available to the field teams. Moreover, the linear geometry of many forested areas (e.g., riparian forests, tree hedges) creates natural movement bottlenecks at our study area (as later observed from the GPS data) which probably increased wildcat-trap encounters. Finally, although the wildcats in NPKV demonstrate the species-characteristic elusive and cryptic nature, the proximity to humans and their activities has rendered them less fearful of traps than other populations. 

The size and inter-sexual differences in wildcat home ranges in Greece agrees with findings from other European populations (males: 1.9–50.2 km^2^; females: 0.7–13.9 km^2^; [19,20,23,31,52,53]). While we did not report overlaps of male and female home ranges, as reported for the species [52] this is likely due to the small sample size of tracked animals in our study. Presence of more individuals within the study area is certain, as confirmed by detections trail cameras during trapping session. The large movement reported by one male in January (and possibly by the second, resulting in us losing track of it), coincided with the onset of the wildcat breeding season (e.g., [23,54]). However, the male shifted its territory during the season, rather than actively patrolling a larger territory during it.

Our observation of the subadult female with a home range size closer to those of males has been previously reported in Italy [31]. As suggested by these authors, this female subadult behavior is likely due to a quest for an unoccupied home-range for their first gestation and parturition. The observed reduction in adult female home range size during spring and early summer has been previously reported as well [55], though it is not a universal pattern [31,56]. This period coincides with female wildcats denning period, during which caring for the offspring keeps them closer to the den. The diel activity change of this adult female from crepuscular/nocturnal to more cathemeral during April–June supports this interpretation. On the other hand, the out of season breeding event of the second adult female, Aphrodite seems to support the breeding pattern that has been recorded in captive animals and suggests that late litters were mostly from second gestations (following the loss of the first litter of that year) and females in their first gestation [54]. Given that the individual was a young adult, the late breeding event in our study can be explained by any of the two reasons mentioned above. 

Overall, the nocturnal activity with crepuscular peaks observed in the two males and one adult female (Ira, during the non-denning period) in NPKV agrees with most studies [23,24,31,52,56]. The cathemeral diel activity of the other adult female, Aphrodite, is more akin to the one of Ira during her presumed denning period (Figure 5a,b). Since Aphrodite was lactating in October, her activity pattern in the first period could be explained by her care for non-weaned kittens. However, this does not explain why her activity remained the same for the rest of the study period. 

It is noteworthy that our findings on wildcat diel activity are based on data of unprecedented temporal resolution: the accelerometer logged activity at 2 min intervals (using 4 sec burst measurements). In contrast, activity patterns deduced from traditional telemetry data are limited to a few dozen records per species or individuals. For instance, Germain et al. [23], using VHF telemetry reported wildcat activity and pause at 4 h time periods. GPS telemetry upgraded the resolution at the level of the location settings (15 min to 3–4 h; e.g., [28]). In just the last few years, technological advances in accelerometer loggers have enabled the kind of data and analyses presented here. Therefore, it is logical to assume that some of the differences of our findings with earlier studies to be at least in part due to differences in the temporal resolution of the data rather than solely to behavioral differences across populations. We anticipate that accelerometer loggers will bring similar changes to diel activity studies of wildlife as GPS telemetry did in home range estimators, leading from the now deemed “crude” MCP to the more refined kernel and Brownian bridge estimators, among others. 

The positive relation of minimum daily temperature and wildcat activity (mean ODBA), especially during nighttime, has not been reported before. In males, this pattern is also true for mean nightly activity and day length, unlike females, where the trend is negative. One possible explanation is that during warmer winter nights, prey may become more active, leading to increased hunting activity by both sexes. In our study area, murids are most likely the primary prey for wildcats (DM, personal observations). There are no rabbits in NPKV; a preferred prey in other wildcat populations [24]. It is unclear why in females, the increased activity during warmer nights ceases as day length increases but persists in males. This behavior could be related to a shift to more diurnal prey (for the female which, during the denning period switched to cathemeral activity) or to a general preference for year-round different prey (for the female with the constantly cathemeral activity). More field studies are required to test the veracity of these hypotheses.

Moonlight illumination varies by three orders of magnitude over a course of a month [57] According to two contrasting hypotheses, small carnivores, like wildcats, which can be both predators and prey, can adapt their behavior in response to available moonlight illumination (and therefore the moon phase) in two ways. Either become more active at bright nights (lunarphilia) since they are visual predators (supporting the “Visual Acuity Hypothesis”) or decrease their activity (lunarphobia) to reduce predation risk (supporting the “Predation Risk Hypothesis”) [58]. Our evidence does not support either of these hypotheses at the level of mean nighttime activity. Studies in tropical felids, show no effect of the moon on their activity [22,59,60], except for lunarphobia in some medium-sized species [58,61]. However, in the tropics, annual variations in day length are minimal compared to the temperate zone, where the European wildcat lives. Potocnik et al. [30], suggested that in Slovenia, there is evidence of increased wildcat activity near full moon. It is possible that in some areas wildcats may show lunarphobia or lunarphilia, in relation to differences in prey and predator community composition. Further field work is required to elucidate these trends in wildcat behavior. 

Adequate resting sites are an important issue in wildlife conservation [62]. Wildcats seem to use a similar number of resting sites per animal in wetland-agricultural mosaics when compared to the ones used in forests for an equal time-period, despite the lower habitat availability. Preserving adequate resting sites must be of high priority for species conservation, when considering management actions.

Finally, when examining non-resting periods, our wildcats did not show any differences in mean activity in areas under or near forest cover and in open areas (fields). At times, wildcats spent hours in open fields, most likely hunting. Along with the spatial data showing clusters of activity in fields, our study showed that in human dominated landscapes, suitable habitat for wildcats is not limited to natural areas but also to the rodent-rich agricultural fields. Crop identification in the most frequently used fields is needed, as their cultivation in agricultural landscapes could be used as a conservation tool for the species.

## 5. Conclusions

The current study is a first step towards filling the current knowledge gap on the spatial ecology and activity patterns of the European wildcat in a landscape heavily modified by humans in Greece and the Balkans in general. This is also the first study to examine wildcat diel activity using the very high temporal resolution data logged by accelerometers. We believe that the combination of these two technologies (GPS + accelerometer) will enable researchers to explore new aspects, not only of wildcat ecology but also of other carnivores and wildlife in general, that were impossible until now, and revisit older ones. Key to this will be the development of supervised or unsupervised, through artificial intelligence (AI) techniques, classifications of accelerometer data, so as to be able to deduce not only overall activity, but also specific behaviors (such as resting, grooming, walking, stalking, pouncing, running, etc. e.g., [63,64]). Ideally, these efforts should be coordinated to include data from wild individuals across the species’ range, so that the developed algorithms have universal applicability. Animals in captivity can also be used in this effort (e.g., see [65]). Since trapping and fitting telemetry and bio-logging sensors to cryptic, low-density species, like the wildcat, will remain expensive in the near future, we propose broad international collaborations that will share both the cost and the effort for developing and testing these behavioral algorithms. 

## Figures and Tables

**Table 1:** Summary table of the tracking period and collected data (GPS locations and accelerometer 2-min logs) for the monitored wildcats.

Animal	Sex	Age (Years)	Weight (kg)	Capture Date	Last Relocation Date	Days Tracked	GPS Fixes	2-min Acc/Meter Logs
Apollo	M	2–3	2.9	29 September 2020	5 January 2021	99	3976	70,190
Aphrodite	F	2–3	3.6	1 October 2020	18 June 2021	261	11,951	170,893
Ares	M	2–3	2.6	2 October 2020	1 June 2021	243	11,845	167,349
Artemis	F	1–2	2.7	30 October 2020	23 June 2021	237	11,278	-
Ira	F	3–4	3.7	4 November 2020	30 June 2021	239	11,875	171,679

**Table 2:** Home range size (in km^2^) of wildcats estimated using the 95% Brownian Bridge (BB) and 95% Minimum Convex Polygon (MCP) home range estimators. Estimates are presented for the total study period and per calendar trimester.

		Aphrodite	Artemis	Ira	Apollo	Ares	Mean
TOTAL	BB	0.94	3.08	0.97	1.22	4.43	2.13 ± 1.40
	MCP	1.69	4.79	1.77	2.35	58.37	13.79 ± 22.32
October–December	BB	1.17	1.10	1.13	1.54	1.64	1.32 ± 0.23
	MCP	2.10	1.12	1.43	2.07	3.73	2.09 ± 0.90
January–March	BB	0.50	2.01	0.87	0.51 ^1^	3.08	1.62 ± 1.012
	MCP	0.44	5.01	0.96	0.83 ^1^	37.87	11.07 ± 15.572
April–June	BB	0.84	1.90	0.67	-	2.07	1.37 ± 0.622
	MCP	1.20	3.31	0.88	-	8.15	3.39 ± 2.912

^1^ Data only from 5 days in January.

**Table 3:** Model averaged estimates and significance of the environmental variables predicting wildcat 24 h activity, as measured in ODBA (daily mean of hourly averages).

Variables	Estimate	SE	z-Value	Pr(>|z|)
intercept (β_ο_)	3963.3	207.0	19.122	<0.0001
day length	−2086.8	321.4	6.484	<0.0001
moon phase	89.6	58.8	1.522	0.128
min. temperature	20.2	4.5	4.463	<0.0001

**Table 4:** Model estimates and significance of the environmental variables predicting wildcat nightttime activity, as measured in ODBA (daily mean of hourly averages) (R^2^ = 0.219).

Variables	Estimate	SE	df	t-Value	Pr(>|z|)
intercept (β_ο_)	4582.1	395.1	22.918	11.598	<0.0001
day length	−2814.1	690.9	797.93	−4.073	<0.0001
min. temperature	81.2	9.7	796.819	8.333	<0.0001

**Table 5:** Number of unique resting sites per month and the duration of the study period for each of the four wildcats with accelerometer.

Individual	Sex	Unique Sites	October	November	December	January	February	March	April	May	June
Aphrodite	F	23	5	4	4	4	6	4	5	2	-
Ira	F	19	-	5	3	6	3	5	4	1	3
Apollo	M	9	5	1	3	-	-	-	-	-	-
Ares	M	23	4	4	4	4	3	5	5	3	-

## Data Availability

Data may be available upon request to the authors.

## Supplementary Materials

The following are available online at www.mdpi.com/article/10.3390/ani11113030/s1, Figure S1: Response curve of mean 24 h and nighttime activity of wildcats to minimum daily temperature, Figure S2: Response curve of mean 24-h and nighttime activity of wildcats to day length, Table S1: AICc model selection of the global model log(ODBA)= βο + daylength + moon phase + min temperature, where ODBA refers to mean 24 h wildcat activity, Table S2: AICc model selection of the global model log(ODBA) = β_ο_ + daylength + rain + min temperature, where ODBA refers to mean nightime wildcat activity, Table S3: AICc model selection of the global model log(ODBA) = β_ο_ + % forest + % forest^2^ + darkness + %forest*darkness, where ODBA refers to mean nightime wildcat activity Table S4: Model estimates and significance of the environmental variables predicting wildcat at non-resting points, as measured in ODBA.
