# Supplementary material for: Spatial Ecology and Diel Activity of European Wildcat (Felis silvestris) in a Protected Lowland Area in Northern Greece
Source: Animals (Basel). 2021 Oct 21;11(11):3030. doi: 10.3390/ani11113030 (PMC8614438; doi:10.3390/ani11113030)
Supplement: Supplementary file 1 [file animals-11-03030-s001.zip › New folder/Supplementary Figures.pdf]

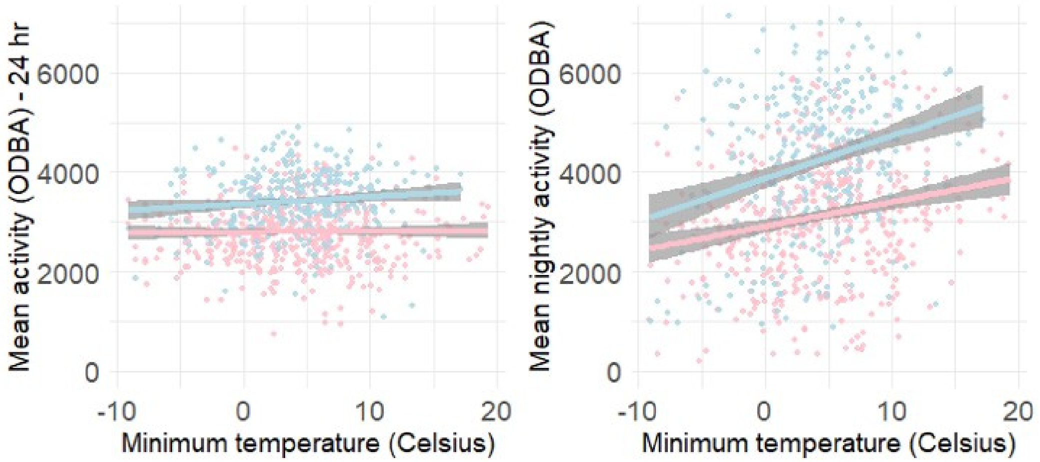

Figure S1. Response curve of mean 24-hr (left) and nighttime (right) activity, as measured in ODBA value, of male (blue) and female (pink) wildcats to minimum daily temperature (shaded areas show the 95% confidence intervals).

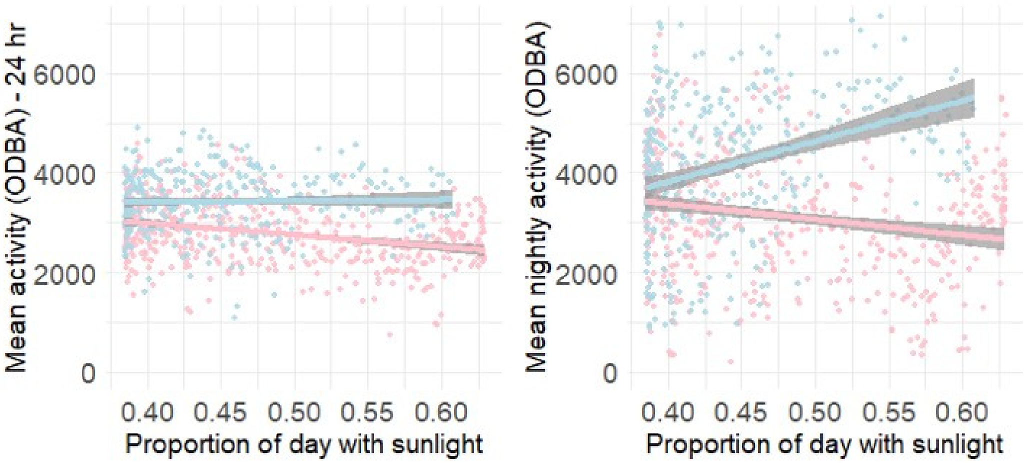

Figure S2. Response curve of mean 24-hr (left) and nighttime (right) activity, as measured in ODBA value, of male (blue) and female (pink) wildcats to day length (i.e. proportion of day with sunglight) (shaded areas show the 95% confidence intervals).

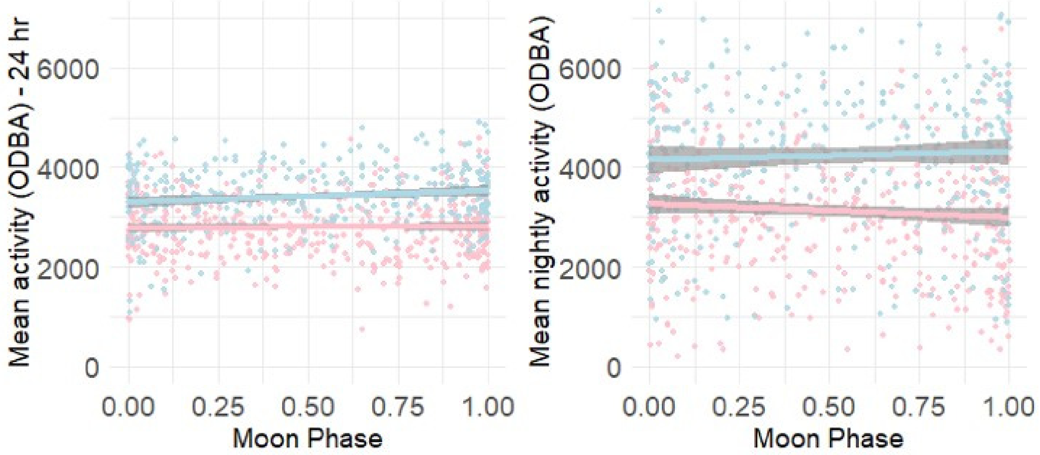

Figure S3. Response curve of mean 24-hr (left) and nighttime (right) activity, as measured in ODBA value, of male (blue) and female (pink) wildcats to the phase of the moon (0 no moon – 1 full moon) (shaded areas show the 95% confidence intervals).
