# Supplementary material for: Spatial Ecology and Diel Activity of European Wildcat (Felis silvestris) in a Protected Lowland Area in Northern Greece
Source: Animals (Basel). 2021 Oct 21;11(11):3030. doi: 10.3390/ani11113030 (PMC8614438; doi:10.3390/ani11113030)
Supplement: Supplementary file 1 [file animals-11-03030-s001.zip › New folder/Supplementary Tables.pdf]

Table S1: AICc model selection (top 5 models + null model) of the global model log(ODBA)= βo + daylength + moon phase + min temperature, where ODBA refers to mean 24-hr wildcat activity. The models included in the final model average are marked in bold.

Model notation	df	LogLik	AICc	ΔAICc	AICw
**log(ODBA)= β_o + daylength + moon phase + min temperature**	***6***	***-6229.43***	***12471***	***0***	***0.536***
**log(ODBA)= β_o + daylength + min temperature**	***5***	***-6230.59***	***12471.3***	***0.29***	***0.464***
log(ODBA)= β_o + daylength + moon phase	*5*	*-6239.29*	*12488.6*	*17.69*	*0*
log(ODBA)= β_o + daylength	*4*	*-6240.46*	*12489*	*18*	*0*
log(ODBA)= β_o + moon phase + min temperature	*5*	*-6249.75*	*12509.6*	*38.61*	*0*
log(ODBA)= β_o (null model)	*3*	*-6252.74*	*12511.5*	*40.54*	*0*

Random effect (individual animal) was kept identical in all GLMM models.

Table S2: AICc model selection (top 5 models + null model) of the global model log(ODBA)= β_o + daylength + rain + min temperature, where ODBA refers to mean nightime wildcat activity (6:30pm – 6:30am). The models included in the final model average are marked in bold.

Model notation	df	LogLik	AICc	ΔAICc	AICw
**log(ODBA)=β_o + day length + min temperature**	***5***	***-6841.76***	***13693.6***	***0***	***0.733***
**log(ODBA)=β_o + day length + rain + min temperature**	***6***	***-6841.76***	***13695.6***	***2.03***	***0.266***
log(ODBA)=β_o + min temperature	*4*	*-6849.96*	*13708*	*14.37*	*0.001*
log(ODBA)=β_o + rain + min temperature	*5*	*-6849.36*	*13708.8*	*15.21*	*0*
log(ODBA)=β_o + precipitation	*4*	*-6872.53*	*13753.1*	*59.52*	*0*
log(ODBA)=β_o (null model)	*4*	*-6875.05*	*13758.2*	*64.56*	*0*

Random effect (individual animal) was kept identical in all GLMM models.

Table S3: AICc model selection (top 5 models + null model) of the global model log(ODBA)= β_o + % forest + % forest^2 + darkness + %forest*darkness, where ODBA refers to mean nightime wildcat activity (6:30pm – 6:30am). The best model used is marked in bold.

Model notation	df	LogLik	AICc	ΔAICc	AICw
**log(ODBA)=β_o + % forest + % forest^2 + darkness + % forest*darkness**	**7**	**-261520.4**	**5230054.8**	**0**	**1**
log(ODBA)=β_o + % forest + darkness + % forest*darkness	6	-261561.8	523135.6	80.81	0
log(ODBA)=β_o + % forest + % forest^2 + darkness	6	-261568.1	523148.3	93.51	0
log(ODBA)=β_o + % forest + darkness	5	-261604.8	523219.6	164.77	0
log(ODBA)=β_o + darkness	4	-261614.3	523236.6	181.78	0
log(ODBA)=β_o (null model)	3	-262313/1	524632.2	1577.39	0

Random effect (individual animal) was kept identical in all GLMM models.

Table S4: Model estimates and significance of the environmental variables predicting wildcat at non-resting points , as measured in ODBA (mean ODBA value for a period of 15 min before and after the GPS record)) (R^2=0.074)

Variables	Estimate	SE	df	t-value	Pr(>|z|)
intercept (β_o)	3610.0	156.2	4.324	23.117	<0.0001
% forest	-2704.7	212.4	27614.361	-12.736	<0.0001
% forest^2	2351.8	258.1	27708.222	9.112	<0.0001
darkness	872.2	39.6	28241.937	22.023	<0.0001
% forest*darkness	1444.8	147.7	28241.097	9.781	<0.0001
